# Estimating the Need for Sedation in Patients with Dental Anxiety and Medical Complexities Reporting to Tertiary Care Dental Hospital Using the IOSN Tool

**DOI:** 10.1155/2022/5824429

**Published:** 2022-04-26

**Authors:** Beenish Abbas, Ayesha Maqsood, Syeda Rabia Rahat Geelani, Madeeha Sattar, Majida Rahim, Zohaib Khurshid

**Affiliations:** ^1^Department of Pedodontics, Foundation University College of Dentistry, Islamabad, Pakistan; ^2^Department of Oral and Maxillofacial Surgery, Foundation University College of Dentistry, Islamabad, Pakistan; ^3^Department of Operative Dentistry, Islamabad, Pakistan; ^4^Department of Oral Medicine, Foundation University College of Dentistry, Islamabad, Pakistan; ^5^Department of Prosthodontics and Dental Implantology, College of Dentistry, King Faisal University, Al-Ahsa 31982, Saudi Arabia

## Abstract

**Objectives:**

To provide consistent method for assessment of sedation need among patients undergoing dental treatment based on specific risk factors that is dental anxiety, medical status, and treatment complexity of needed dental treatment using IOSN (indication of sedation need) tool for assisting the clinician in decision making process.

**Methods:**

A total of 237 patients aged ≥12, ASA I and II were enrolled in the study. A structured questionnaire comprising of three sections was distributed among the participants. Section 1 comprises details about age, gender, literacy level, occupation, monthly income, and previous dental treatment history. Section 2 is based on Modified Dental Anxiety Scale which is a questionnaire comprising of five questions ranging from “not anxious” to “extremely anxious.” The third section was based on using the IOSN tool comprising three components: MDAS (Modified Dental Anxiety Scale) rank score, Systemic Health (ASA status) rank score, and treatment complexity rank scores. The total of three scores was then computed to determine the total rank score which suggested the sedation need. History of past traumatic dental experiences was also inquired from each patient.

**Results:**

A total of 237 patients aged ≥12, ASA I and II were enrolled in the study, out of which 56.1% were female. Statistical analysis was conducted by using the IBM SPSS Statistics 23 software. Based on the MDAS score, 47/237 (19.8%) participants were found to be highly anxious related to dental procedures. 34.6% of the participants showed to have a high sedation need while performing a dental procedure. The sedation need was found to be significantly associated with the female gender with a significant *p* value of (*p*=0.016), higher education status (*p*=0.016), and history of previous traumatic dental experience (*p* < 0.001).

**Conclusion:**

A simple assessment tool can enable clinicians in their decision making to identify patients in need for dental treatment under sedation based on patient-specific risk factors such as past traumatic dental experiences. Need for sedation can be assessed by information on patient anxiety level towards dental treatment, medical history, and complexity of planned dental treatment. The IOSN tool is a simple and quick assessment tool that can be applied for preprocedural assessment of sedation need for dental treatment.

## 1. Introduction

Vicarious learning from anxious peers, past traumatic dental experiences, and certain personality traits contribute towards the development of lifelong behavior issues such as dental anxiety and phobias which may necessitate the need for sedation. Sedation is also indicated for patients reporting with medical conditions which may aggravate under a stressful environment like in angina patients, anxiety and pain can increase the release of endogenous catecholamines, thereby increasing the load on the cardiovascular system. Similarly, asthmatic patients can present with an acute episode of breathing difficulty induced by stress [1].

Sedation is a safe and effective pharmacological behavior management modality which can be used as an alternative to more complex options such as general anaesthesia in certain patients. In this pharmacological anxiety reduction protocol, use of drugs causes depression of the central nervous system enabling treatment to be carried out in a more relaxed environment. At the same time, verbal contact with the patient is maintained throughout treatment [1].

According to the treating clinician and patient perspective, sedation for complex dental treatment has been gaining popularity in recent years, as patients are more informed about the anxiety reduction treatment options available. However, adhering to strict standard clinical screening and individualized patient treatment needs is essential to ensure good clinical practice [2]. To achieve this, there is a need for an objective, comprehensive assessment tool to evaluate the need for sedation as clinical decision making is complicated. The tool should be useful in selecting the right patient based on medical history, level of dental anxiety, and complexity of dental treatment needed. This aids in optimizing patient safety and setting guidelines for adequate staff training [3].

This study aims to work on a simple assessment tool to provide the minimum patient data set needed to establish the need for sedation. Sociodemographic factors, past traumatic dental experiences, and dental anxiety will be assessed using a questionnaire based on a modified dental anxiety scale. The American Society of Anesthesiologist classification system will be used to assess the medical status of the patient, and each patient will be assigned a score to grade dental treatment complexity level. This novel assessment tool does not take more than a few minutes of treating clinicians' time to provide comprehensive patient assessment.

## 2. Methodology

The study was conducted after approval from the Ethical Review Committee of the Foundation University College of Dentistry, Islamabad, with ERC ref no. (FF/FUCD/632/ERC001).

The minimum sample size required for this prospective cross-sectional analytical study was 237, calculated by using formula (*n* = [deff *∗* np(1-p)]/[(d2/*z*21-*α*/2 *∗* (n-1)+*p∗* (1-p)]-open epi calculator), with 95% confidence level and 5% margin of error [4]. A nonprobability consecutive sampling methodology was employed and total (*n* = 250) participants enrolled in the study. Informed written consent was obtained with the assurance of anonymity and confidentiality from the participants before filling out the questionnaire.

Patients aged >12 years, irrespective of gender, were recruited in the study. Nonconsenting participants, mentally incapacitated patients with debilitating mental or physical illness (ASA 3 and above) and/or already on any psychiatric or chronic ailment treatments and patients with a history of insomnia who were on sedatives were excluded from the study.

A structured questionnaire comprising of three sections was distributed among all the study participants. The questionnaire was designed both in Urdu and English after validation and expert opinion on the subject. Section 1 (anxiety questionnaire to be completed by patient) comprised the details such as patient age, gender, occupation, monthly income, and past experience of traumatic dental treatment. Section 2 is based on Modified Dental Anxiety Scale which is a form of questionnaire comprising of five questions in which range of each item is from “not anxious” to “extremely anxious” assessed on following occasions:

- Dental clinic visits.
- While in the waiting room for dental treatment at the dentist's office.
- Awaiting in the dental chair for tooth drilling.
- Awaiting in the dental chair for teeth scaling.
- Awaiting in the dental chair for local anesthetic infiltration.

**Figure 1:**
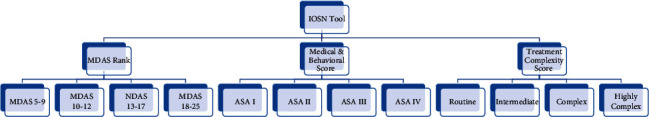
IOSN tool.

Not anxious is given a score 1 and highly anxious is given a score 5. The modified dental anxiety scale questionnaire which is used to assess anxiety component of IOSN tool provides the summed score between 5 to 25 which is then converted to rank score of 1—4. ASA I patients were assigned rank score 1, ASA II patients according to their medical condition was rank scored as 2. ASA III patients were allotted rank score 3. Third component of the IOSN tool was related to treatment complexity of needed dental treatment routine procedures such as scaling, and single quadrant restorations were rank scored as 1. Intermediate complexity procedures such as surgical extraction, scaling with root planning, or two quadrant restorations were allotted rank score 2. Complex dental procedures such as periodontal surgery, surgical extraction with bone removal, apicectomy posterior tooth, multiple quadrant restorative, and multiple posterior endodontics. First component of the IOSN tool was MDAS score; modified dental anxiety scale score (5–9) indicating minimal dental anxiety was ranked score as 1, MDAS score (10–12) indicating moderate patient anxiety was given rank score 2. Likewise MDAS score (13–17) indicative of high patient dental anxiety was assigned rank score 3. Similarly, MDAS value of (18–25) showing very high dental anxiety was rank scored as 4. The second component of the IOSN tool comprised patient medical status. Periodontal surgery was assigned a rank score of 3. High complexity dental procedures were allotted rank score 4. For each patient anxiety score, medical history score and treatment complexity score was ranked and entered in the IOSN tool and summative score of all three components of the IOSN tool gives an overall score between 3 and 12 with lowest score 3 indicating minimal need for sedation. (Figure 1)

### 2.1. Indicator of Sedation Need Tool

- Routine: scale, single rooted extraction of 1 or 2 teeth, small soft tissue biopsy, single quadrant restorations, crown preparations, or anterior endodontic treatment.
- Intermediate: scale and root planning, multirooted tooth extraction, surgical extraction without bone removal, apicectomy anterior tooth, 2 quadrant restorative, and posterior endodontic treatment.
- Complex: periodontal surgery, surgical extraction with bone removal, apicectomy posterior tooth, multiple quadrant restorative, and multiple posterior endodontics.
- High complexity: any treatment considered more complex than above or are multiples of the above.

Statistical analysis was performed by the IBM SPSS Statistics 23. Descriptive statistics were performed for age and demographic data. A chi-square test was used to find a statistically significant association between variables. A *p* value of ≤0.05 was considered statistically significant.

## 3. Results

The data from 237 participants were considered for analysis in this study. Out of 237, 104 (43.9%) males and 133 (56.1%) females were in the study group. The majority of the participants, 96 (40.5%) belonged to age group of 41–50 years, while least, 47 (19.8%), were from 21 to 30 years age group. Out of 237, 150 (63.2%) were unemployed including students, housewives, and retired persons, while the remaining 87 (36.7%) were employed. 48 (20.3%) participants were with no education, while 49 (20.7%) were graduates and 56 (23.6%) also had postgraduation. The demographic characteristics of the study participants are summarized in Table 1 in detail.

**Figure 2:**
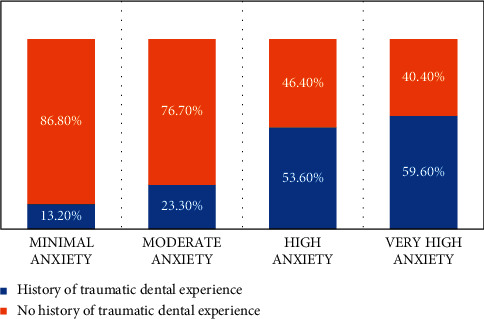
Comparison of mean dental anxiety score (MDAS) classes with past traumatic dental experience.

Experience of traumatic and fearful dental procedures was reported by 90/237 (38.0%) of the participants. The Modified Dental Anxiety Score (MDAS) was calculated to be 12.87 ± 4.9 based on five anxiety assessment questions. Based on MDAS score, 47/237 (19.8%) participants were found to be experiencing very high anxiety related to dental procedures as shown in Figure 2. The dental procedure anxiety was significantly associated with female gender (*p* = 0.016), higher education status (*p*=0.012), and past traumatic/fearful dental procedure (*p* < 0.001). The majority of the participants 127 (53.5%) reported to feel anxious a day before going to the dentist for a dental procedure appointment (grade 2 and above on scale of 1–5). Similarly, significant portion of participants, 133 (56.1%) said to feel anxious while sitting in waiting room before getting a dental procedure done. During the procedure, 189 (79.7%) reported to have felt anxious before getting a tooth drilled (grade 2 and above on scale of 1–5), 136 (57.3%) felt anxious before getting scaling/polishing of tooth, while 202 (85.2%) felt anxious before getting local anaesthesia injection in gum (grade 2 and above on scale of 1–5), as depicted in Table 2.

In terms of medical and behavioral indicator rank score, 141 (59.5%) participants belonged to ASA grade 1, while the remaining 96 (40.5%) belonged to grade 2. Concerning treatment complexity score 24 (10.1%) participants underwent routine procedures including scaling, single rooted extraction of 1 or 2 teeth, small soft tissue biopsy, single quadrant restorations, crown preparations, or anterior endodontic treatment, whereas 191 (80.6%) participants underwent intermediate procedures including scale and root planning, multirooted tooth extraction, surgical extraction without bone removal, apicectomy anterior tooth, 2 quadrant restorative, and posterior endodontic treatment. Complex procedures including complex periodontal surgery, surgical extraction with bone removal, apicectomy posterior tooth, multiple quadrant restorative, and multiple posterior endodontics were performed in only 22 (9.3%) participants.

The mean sedation need score based on mean dental anxiety score (MDSA), medical and behavioral indicator rank score, and complexity of dental procedure score was calculated to be 5.83 ± 1.35. It was found that 49 (20.7%) participants had minimal need of sedation during dental procedure, 106 (44.7%) had moderate need whereas 82 (34.6%) were found to have a high sedation need while performing a dental procedure. The sedation need was found to be significantly associated with female gender (*p* = 0.034) and history of previous traumatic dental experience (*p* < 0.001) as shown in Table 3.

## 4. Discussion

Various studies have been conducted locally and internationally, which evaluate the dental anxiety scale of the patient [5]. Recently published literature supported the need for risk-based preprocedural assessment to make an objective decision of providing sedation. Keeping in view the patient risk factors, preprocedural risk assessment to determine the need for sedation is described as the minimum standard of care as reported by Sutherland et al. [6].

Nowadays, patient-focused healthcare and decision making are given prime importance. That is why the patient's level of anxiety regarding dental treatment will help the dentist reach a decision regarding the need for sedation. The use of sedation enables the patient to have his required dental treatment without finding it stressful [7]. Use of sedation is variable among dentists, with some using it injudiciously, whereas others are not using it at all.

The anxiety component of the IOSN Tool (Indicator of Sedation Need Tool) was calculated using Modified Dental Anxiety Scale (MDAS) as it is deemed as a very relevant and brief tool [8].

Pretreatment compounding factors have positive influence on anxiety levels such as previous dental procedures performed and environmental factors which are divided into three categories including personal factors such as patient age and mental status. Second important environmental factor is external factors such as socioeconomic status, education, and racial factors. Dental factors such as attitude of dental team are also an important environmental factor to consider towards patient anxiety [9]. Similarly, females showed higher level of anxiety while giving local anaesthesia and tooth being drilled compared to males [10]. Another study supported that gender (females), age, and education significantly affect dental anxiety [11]. Similarly, the results of our study also found that dental procedure anxiety was significantly associated with female gender (*p*=0.016).

Dental anxiety is stress-provoking both for patients and dentist alike, leading to misdiagnosis and an unpleasant treatment environment due to diminished patient cooperation which may ultimately lead to poor oral, periodontal health, and increased emergency attendance resulting from avoidance of dental appointments as reported by Zinke et al. [12]. Irregular dental visits leading to poor oral health is associated with dental anxiety and phobias. Younger patients, females, and patients with previous unpleasant dental experience were associated with increased MDAS scores. Likewise, in our study, correlation has been established that 59.60% patients having past traumatic dental experiences presented with very high dental anxiety [13, 14].

Demographic characteristics of our study participants regarding their educational status show somewhat equal distribution of participants regarding their education status, with graduate and postgraduate being slightly high in number. Higher the educational status, more was the anxiety, as seen in this study (*p*=0.012). Similar findings were also observed in other studies [11, 15].

Based on MDAS (Modified Dental Anxiety Scale) score, most of the participants in our study felt anxious while sitting in a waiting room before getting a dental procedure done (grade 2 and above on scale of 1–5), while it was closely followed by 53.5% who felt anxious a day before going to dentist. MDAS was selected to measure the dental anxiety component of the IOSN tool and need for sedation because this scale is simple and universally well accepted [16, 17].

The patients' anxiety depends upon the dental procedure he is undergoing. Our study suggests that quite a high proportion of patients (42.2%) were extremely anxious while getting local anaesthesia. Another study reported higher anxiety scores in patients receiving local anaesthesia [18]. This was contradictory to a study where most of the patients who were having dental filling exhibited most anxiousness [19].

Recent study reported that patients with traumatic dental event have higher scores of dental anxiety (*p* = 0.028) which leads to negative perceptiveness about dental treatment and dentist [20]. Our study has a significant association of dental anxiety with past traumatic dental experience (*p* > 0.001). In terms of traumatic and unpleasant past dental experiences, younger the patient at the time of fearful dental experience, higher are the chances of translating into more anxious behavior owing to the emotional vulnerabilities of adolescents and children. Another study reported significant higher IOSN scores and need of sedation in 60.3% of patients having unpleasant dental experiences. Patients who reported previous traumatic dental experiences were 2.24 times more likely to need sedation [21]. A recent study by Merdad and El-Housseiny shows a significant association between traumatic dental experience and poor oral health-related quality of life had greater caries experience with more avoidance behavior towards dental appointments [22].

While comparing the medical risk factors score, most (59.5%) of the participants belonged to the group ASA Grade I while the remaining (40.5%) belonged to Grade II. Another recent study by Dziedzic et al. highlighted the crucial role of the comprehensive assessment strategy in medically compromised and special care patients for safe delivery of conscious sedation as an alternative to general anaesthesia for vulnerable patient group [23].

In our study, while applying the IOSN tool, 34.6% were found to have a high sedation need while performing the dental procedures. This was contradictory to a study which found that only 2.4% of the patients showed a high need for sedation when the IOSN tool was applied to their study population [24].

More the complexity of dental procedure, more is the need for sedation. That is why we see that in minor oral surgical procedures such as third molar surgery, many of the dentists prefer sedation [25]. Many methods for sedation are being used for third molar impactions. All these methods aim to achieve better patient outcomes in terms of pain control as noted in other studies [26]. Also, patients presenting with periodontal disease with higher anxiety levels, while undergoing scaling and root planning treatments may exhibit negative clinical outcomes. Thus, suggesting that even for simpler procedures sedation can play an important role to achieve better patient outcomes in terms of pain control [27].

Preprocedural comprehensive patient evaluation is an integral part of an up-to-date sedation practice. Detailed evaluation of patient parameters is of utmost significance in developing an individualized agreed-upon sedation plan that reflects minimum standard of dental care with low risk of complications. Research is lacking on the development and application of a validated sedation scoring system to predict requirement of sedation based on individual patient parameters which would further enhance shared decision making between clinician and patients as it holds utmost significance to enhance patient satisfaction [6]. The recent pandemic has led to increasing demands on the hospitals with deferral of general anaesthesia and hospital-based sedation. Therefore, the use of dental sedation is the need of hour to provide optimal dental services in dental clinics on out-patient basis. In order to provide required support in specialized dental care and to meet the current health care needs, we need more trained personnel in the field of sedation [23].

The limitation of this study was a smaller sample size and a wider age range, and the data were recruited from single-hospital setting, thus lacking the generalizability. Similarly, we applied this tool over a variety of the dental procedures, some complex and some rather simple. The study lacks the degree of variability because anxiety questionnaire score revolves around dental treatment events and noncognitive factor (traumatic dental event). This tool can be further modified as it has certain limitations. It does not indicate the level of sedation required for a particular patient, ranging from mild sedation to general anaesthesia. This study was an initial step towards applying this innovative tool for calculating the need for sedation in dental patients. In future, this could be used to carry out multicenter studies which will help in further validation of this tool. This instrument could be modified and applied for patients with special needs and pediatric patients.

## 5. Conclusion

A simple assessment tool can enable clinicians in their decision making to identify patients in need for dental treatment under sedation based on patient-specific risk factors such as past traumatic dental experiences. Need for sedation can be assessed by information on patient anxiety level towards dental treatment, medical history, and complexity of planned dental treatment.

## Figures and Tables

**Table 1:** Demographic characteristics of study participants (*n* = 237).

Demographic characteristics		N (%)
Age groups	12–20 years	44 (22.3%)
	21–30 years	47 (19.8%)
	31–40 years	50 (21.1%)
	41–50 years	96 (40.5%)

Gender	Male	104 (43.9%)
	Female	133 (56.1%)

Occupation	Student	66 (33.3%)
	Housewife	82 (34.5%)
	Private job	40 (16.9%)
	Government job	25 (10.5%)
	Self-employed	22 (9.3%)
	Retired from army	2 (1.0%)

Monthly income (PKR)	Dependent	150 (63.3%)
	20–30,000	22 (9.3%)
	31–50,000	27 (11.4%)
	>50,000	38 (16.0%)

Education	Less than primary	48 (20.3%)
	Primary	46 (19.4%)
	Secondary	38 (16.0%)
	Graduate	49 (20.7%)
	Postgraduate	56 (23.6%)

**Table 2:** Frequency of participant responses to questions related to anxiety related to dental procedure (*n* = 237).

	Participant responses				
	1	2	3	4	5
Before going to dentist for treatment tomorrow, how do you feel?	110 (46.4%)	65 (27.4%)	44 (18.6%)	9 (3.8%)	9 (3.8%)
While sitting in waiting room before treatment, how do you feel?	104 (43.9%)	54 (22.8%)	53 (22.4%)	13 (5.5%)	13 (5.5%)
Before getting a tooth drilled, how do you feel?	48 (20.3%)	40 (16.9%)	35 (14.8%)	71 (30.0%)	43 (18.1%)
Before getting tooth scaled and polished, how do you feel?	101 (42.6%)	34 (4.3%)	59 (24.9%)	25 (10.5%)	18 (7.6%)
Before getting local anaesthesia in gum, how do you feel?	35 (14.8%)	34 (14.3%)	33 (13.9%)	35 (14.8%)	100 (42.2%)

Participant responses: 1, not anxious; 2, slightly anxious; 3, fairly anxious; 4, very anxious; 5, extreme anxious.

**Table 3:** Sedation need while performing dental procedure and its association with past traumatic dental experience.

	Categories of sedation need			*P* value
	Minimal (*n* = 49)	Moderate (*n* = 106)	High (*n* = 82)	
Gender				
Male	15 (30.6%)	45 (42.5%)	44 (53.7%)	**0.034**
Female	34 (69.4%)	61 (57.5%)	38 (46.3%)	

Past traumatic dental experience				
Yes	1 (2.0%)	47 (44.3%)	42 (51.2%)	**<0.001**
No	48 (98.0%)	59 (55.7%)	40 (48.8%)	

Bold shows *P* value of less than .001 is considered statistically significant.

## Data Availability

The data used to support the findings of this study are available on request.
